# The Surgery of the Spleen and Pancreas

**Published:** 1905-02-11

**Authors:** 


### THE SURGERY OF THE SPLEEN AND PANCREAS.

Primary Sarcoma of the Spleen.—Jepson and Albert[1] point out that a diagnosis at an early stage, before the occurrence of metastases, is essential to the successful treatment of the disease by splenectomy. The early recognition of malignant growths of the spleen presents some difficulties on account of the meagre symptoms which result from its presence. We could only expect the presence of three symptoms, namely—(1) enlargement of the spleen. (2) Pain. (3) Blood changes. The first symptom, viz., enlargement, must be difficult of recognition in the early stage, owing to the protected position of the spleen making palpation impossible, while percussion is not to be relied upon. Owing to the only moderate support which the spleen has through the reflections of the peritoneum, it seems that the increased weight soon lengthens these attachments, with the result that the spleen at an early period acquires a range of mobility making it possible for it to descend below the costal margin, where it may become palpable, thus giving us the most trustworthy evidence possible of attainment in the light of our present knowledge of the subject. It having been determined that the growth pertains to the spleen, the nature of the enlargement will present itself for solution. The physical characteristics of the growth will largely aid in this. Thus, if the spleen is uniformly enlarged, with its normal shape well maintained, it would be a justification for excluding all primary neoplasms, at least in the early stage of their formation. While if, on the other hand, the spleen is not uniformly enlarged, but the seat of nodosities, or localised enlargement, it would bring up for review those various conditions capable of bringing this about. If it is possible to determine by palpation or aspiration that the enlargement is solid, then, of course, there is excluded echinococcus and other cysts as well as abscess of the spleen. The growth being a solid one, the question of its being a sarcoma, fibroma, angioma, or lymphangioma appears for solution; and this must certainly be at times quite impossible until the growth is exposed to inspection by exploratory incision. Rapid growth does not necessarily occur in cases of sarcoma, and in any case if malignancy is suspected it would seem unwarrantable to defer treatment until the rapidity of growth could be definitely determined. Considerable pain would be expected to exist owing to distension of the splenic capsule, yet this does not seem to be a symptom of much value, and it is often absent, and if present may be mistaken for renal or gastric pain. Blood examination seems to be of no real value from a diagnostic standpoint. It seems, therefore, clear that with evidence of a solid growth of the spleen a definite diagnosis cannot be made until the organ is exposed by means of an exploratory incision. As to treatment, no question can exist as to the advisability, in the absence of secondary involvement of other organs, of removing the spleen, which in the hands of experienced surgeons must be accompanied with but a small mortality. Of 11 splenectomies three died following the operation.

**Glandular Cyst of the Pancreas** according to Gourand [2] is rather rare. It is an affection of adults, being most frequently found between the ages of 25 and 45. Trauma is a frequent cause, and the author found that injury seemed to be the starting point of the cyst in 16 out of 96 cases. Nearly always the trouble begins insidiously with troubles of digestion, slight weakness, and vague pains in the left hypochondriac region. It is during the first period of very variable duration when diagnosis is impossible. When the tumour appears it grows rapidly, and the functional symptoms increase, while the phenomena of compression begin. At this time diagnosis and intervention are possible. If, however, intervention is not made, there develop cachexia, and vascular, and digestive troubles which are almost like those of large abdominal tumours. Complications of pancreatic cyst are rare and unimportant. It is a serious malady, and if intervention does not take place death occurs at the end of a year or two. As to operation, one must be guided by the clinical variety of the cyst. Total extirpation is indicated if the cyst is accessible and well pediculated. On the contrary, the surgeon must be content with simple incision and drainage, which almost always brings about cure. Moore [3] records a case of general pancreatitis successfully treated by cholecystenterostomy.

1 Annals of Surg., July 1904, p. 80. 2 Med. Record, May 14, 1904, p. 790. 3 Lancet, July 3, 1904, p. 25.

